# 1522. Predictors of Weight Gain in People Living with HIV

**DOI:** 10.1093/ofid/ofad500.1357

**Published:** 2023-11-27

**Authors:** Karen B H Pedersen, Susanne Dam Poulsen, Thomas Benfield

**Affiliations:** Department of Infectious Diseases, University Hospital Amager and Hvidovre, København N, Hovedstaden, Denmark; Departments of infectious Diseases, Rigshospitalet, Denmark, Copenhagen, Hovedstaden, Denmark; Copenhagen University Hospital, Hvidovre, Hovedstaden, Denmark

## Abstract

**Background:**

Highly effective antiretroviral therapy (ART) and longer life expectancy in people living with HIV (PLWH) has led to increased incidence of lifestyle associated risk factors including overweight and obesity. ART is considered to play an important role in this tendency. Excess weight gain has been reported in PLWH treated with and integrase strand transfer inhibitors (INSTI). For nucleoside/nucleotid reverse transcriptase inhibitor (NRTI) backbone components tenofoviralafenamid (TAF) is associated with higher weight gain compared to tenofovir disoproxil (TDF) and abacavir (ABC). This study aims to investigate ART as predictors of weight gain in a prospective cohort using a target trial emulation.

**Methods:**

The protocol for the target trial was established to evaluate the impact of baseline backbone treatment and various third components on weight gain over a 2-year follow-up period using the Copenhagen Co-Morbidity in HIV Infection Study (COCOMO) cohort. Data was collected from March 2015 to December 2016 in the Capital Region of Denmark. Adult PLWH were included. Subjects were divided into four different treatment groups according to their backbone components: Lamivudine/emtricitabin (XTC)/TDF, XTC/ABC, XTC/TAF or “other”. Regression standardization was applied to the linear regression outcome adjusted for relevant confounders.

**Results:**

In total 704 subject were included in the analysis. Overall median weight gain in the 2-years follow up period was 0.7 IQR[-22.4, 26.0] with the largest weight gain in subjects with XTC/TDF at baseline (median 1.0 IQR[-12.6, 26.0]). Regression standardization of the adjusted linear regression model showed no significant difference in weight gain between backbone groups. In the third-component analysis including subjects with XTC/TDF or XTC/ABC backbone, subjects receiving an integrase inhibitor based regimen at baseline tended to have a 0.75 kg higher weight gain compared to NNRTI- and PI-based regimens, but this was not significant S.E. 0.474, (0.17;1.67 95%CI).

**Conclusion:**

The results suggest that while antiretroviral therapy may contribute to weight gain, the choice of backbone components may not be a significant predictor. Further studies are needed to confirm these findings.

**Disclosures:**

**All Authors**: No reported disclosures

